# Peptidylarginine deiminase 1-catalyzed histone citrullination is essential for early embryo development

**DOI:** 10.1038/srep38727

**Published:** 2016-12-08

**Authors:** Xiaoqian Zhang, Xiaoqiu Liu, Mei Zhang, Tingting Li, Aaron Muth, Paul R. Thompson, Scott A. Coonrod, Xuesen Zhang

**Affiliations:** 1 State Key Laboratory of Reproductive Medicine, Nanjing Medical University, Nanjing, China; 2 Key Laboratory of Pathogen Biology of Jiangsu Province, Nanjing Medical University, Nanjing, China; 3 Department of Microbiology, Nanjing Medical University, Nanjing, China; 4 Department of Biochemistry and Molecular Pharmacology, University of Massachusetts Medical School, Worcester, MA, USA; 5 Baker Institute for Animal Health, College of Veterinary Medicine, Cornell University, Ithaca, NY, USA

## Abstract

Peptidylarginine deiminase (PADI) enzymes are increasingly being associated with the regulation of chromatin structure and gene activity via histone citrullination. As one of the PADI family members, PADI1 has been mainly reported to be expressed in the epidermis and uterus, where the protein in keratinocytes is thought to promote differentiation by citrullinating filament proteins. However, the roles of PADI1 in preimplantation development have not been addressed. Using a PADI1-specific inhibitor and Padi1-morpholino knockdown, we found that citrullination of histone tails at H4R3 and H3R2/8/17 were markedly reduced in the 2- and 4-cell embryos. Consistent with this observation, early embryo development was also arrested at the 4-cell stage upon depletion of PADI1 or inhibition of PADI1 enzyme activity. Additionally, by employing 5-ethynyl uridine (EU) incorporation analysis, ablation of PADI1 function led to a dramatic decrease in overall transcriptional activity, correlating well with the reduced levels of phosphorylation of RNA Pol II at Ser2 observed at 2- or 4-cell stage of embryos under Padi1 knockdown or inhibiting PADI1. Thus, our data reveal a novel function of PADI1 during early embryo development transitions by catalyzing histone tail citrullination, which facilitates early embryo genome transactivation.

During preimplantation development, mammalian embryos undergo robust and dynamic changes in their gene expression patterns^1^,^2^,^3^,^4^. In the mouse, transcription from the newly formed zygotic genome, also known as embryonic genome activation (EGA), mainly occurs at the 2-cell stage. This first major transition promotes the generation of novel transcripts that are not expressed in oocytes^5^,^6^,^7^,^8^,^9^. A second wave of new gene transcripts, named mid-preimplantation gene activation, appears between the 4-cell and 8-cell stages, and are involved in the beginning of compaction^2^,^4^,^10^,^11^, followed by the dynamic morphological and functional changes from the morula to blastocyst stage^5^,^12^. Though accumulating efforts have been focused on identifying maternal and embryonic transcripts involved in early embryo development, the potential underlying factors controlling these two wave transitions still remain to be explored.

Peptidylarginine deiminases (PADIs) are a family of calcium dependent enzymes that convert positively charged arginine residues to citrulline via a hydrolytic process termed citrullination or deimination^13^. This post-translational modification alters protein charge resulting in changes in protein structure, function, and molecular interactions^14^. There are five PADI family members, PADI1-4 and PADI6. Each PADI enzyme has a unique tissue distribution pattern and distinct substrate specificity, except for PADI6 which does not appear to have catalytic activity^15^. PADI-mediated histone citrullination is increasingly being associated with the regulation of chromatin structure and gene activity. For example, we recently found that PADI2-catalyzed histone H3 arginine 26 citrullination leads to chromatin decondensation and transcriptional activation in human breast cancer MCF7 cells^16^. PADI2 also targets histone H3 arginine 2/8/17 for citrullination in mammary epithelial cells and appears to regulate the expression of lactation related genes in mammary epithelial cells^17^. PADI4 regulates the proliferation of multipotent progenitors in the bone marrow by controlling c-myc expression through catalyzing the citrullination of histone H3 on its promoter^18^. In addition to histone H3 citrullination, PADI4-catalyzed histone H4 arginine 3 citrullination at the *TFF1* promoter in MCF7 cells appears to regulate its expression^19^. Citrullination of histone H1 mediated by PADI4 at promoter elements can also promote localized chromatin decondensation in stem cells, thus regulating the pluripotent state^20^. These observations suggest that PADI-mediated histone citrullination profoundly affects chromatin structure and regulates chromatin-based activities.

Interestingly, a potential role for histone citrullination in chromatin-based activities during preimplantation development has also been investigated^21^. In that report, the specific arginine residues on the histone H3 and H4 N-terminal tails (H4R3, H3R2/8/17) were citrullinated and showed a unique localization pattern during early development. Inhibiting histone citrullination at the pronuclear stage zygotes blocked embryonic development beyond the 4-cell stage. However, it was not clear which PADI catalyzes histone tail citrullination in preimplantation development and the underlying mechanism is far from complete. In this study, by employing a PADI1-specific inhibitor and morpholino knockdown, we showed that inhibition of PADI1 activity and depletion of PADI1 suppress histone tail citrullination and genome transactivation in mouse early embryos, thus leading to preimplantation development arrest at 4-cell stage.

## Results

### Cellular localization of PADI1 in mouse oocyte and during preimplantation embryo development

**Figure 1:**
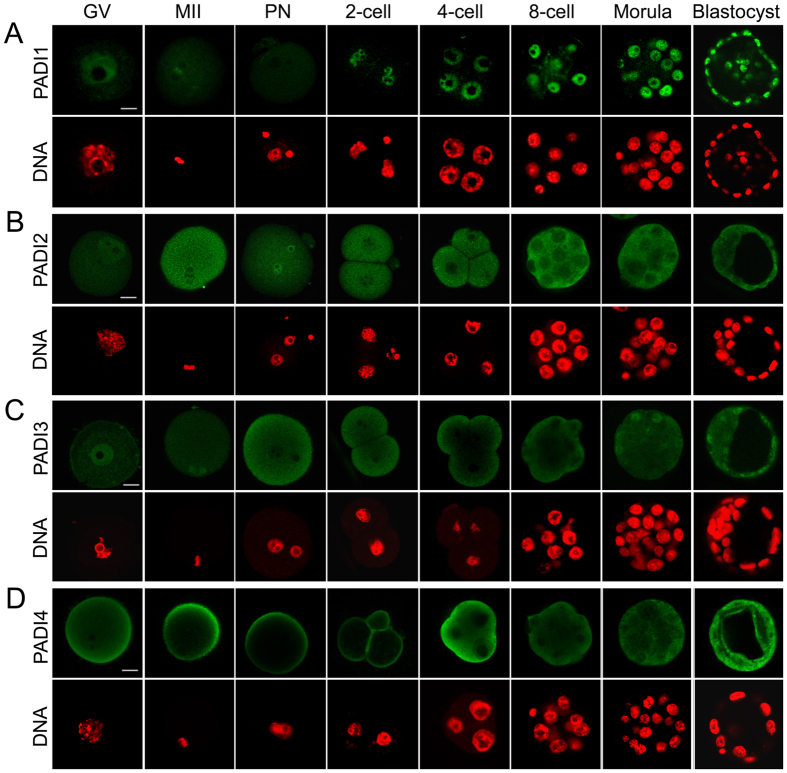
Immunofluorescence staining of PADI1, PADI2, PADI3, and PADI4. Mouse oocytes at GV, metaphase II, and preimplantation embryos at different stages (1-cell embryos (PN), 2-cell embryos, 4-cell embryos, 8-cell embryos, morula, and blastocyst) were immunostained with antibodies specific for PADI1 (**A**), PADI2 (**B**), PADI3 (**C**), and PADI4 (**D**), respectively, and counterstained with DAPI for nuclear staining (red). Scale bar, 20 μm.

To look for which PADI family member may mediate histone tail citrullination in early embryo development, we first analyzed the expression pattern of PADI1 in mouse oocytes and throughout early embryo development by immunofluorescent staining coupled with confocal microscopy. As shown in Fig. 1A, PADI1 protein was predominantly localized in the nuclei of germinal vesicle (GV) and metaphase II (MII) oocytes. With a gradual decrease of expression in pronuclear (PN) stage of zygotes, PADI1 started to accumulate dramatically in the nuclei of embryos from the 2-cell stage, and this intense fluorescent staining for PADI1 was observed in the nuclei of 4-cell embryos, 8-cell embryos, morula, and blastocyst. This expression pattern indicated that PADI1 might play an important role in early embryo development, especially starting at 2- or 4-cell stage. To further confirm the anti-PADI1 antibody specificity, an overexpressed Flag-tagged or His-tagged version of PADI1 was readily detected in HEK293 cell extracts by western blot using anti-PADI1 antibody, while this antibody does not recognize other PADI family members in the lysates of HEK293 cells that had been transfected with Flag-PADI2, Flag-PADI4, or His-PADI3 tagged expression vectors (Supplementary Fig. S1). We also examined the expression and localization of the other PADI family members in mouse oocytes and early embryos during all stages of development. Unlike PADI1, expression of PADI2, PADI3, or PADI4 protein mainly localized to the cytoplasm of the oocytes and preimplantation embryos (Fig. 1B–D), with only slight immunofluorescence signals for PADI2 and PADI3 observed in the nuclei of GV oocytes (Fig. 1B,C), and occasional weak nuclear expression of PADI3 in the morula and blastocyst (Fig. 1C). PADI6 was not tested here given that PADI6 only localizes in the cytoplasm of germ cells and embryos, and PADI6 does not have enzyme catalytic activity^15^. These results suggest that PADI1 may function as a predominant nuclear PADI in mouse preimplantation embryos. Such a distribution pattern of PADI1 prompted us to explore its potential roles in mouse oocyte maturation and during preimplantation embryo development.

### Inhibiting PADI1 enzyme activity or depletion of PADI1 compromises early embryo development

**Figure 2:**
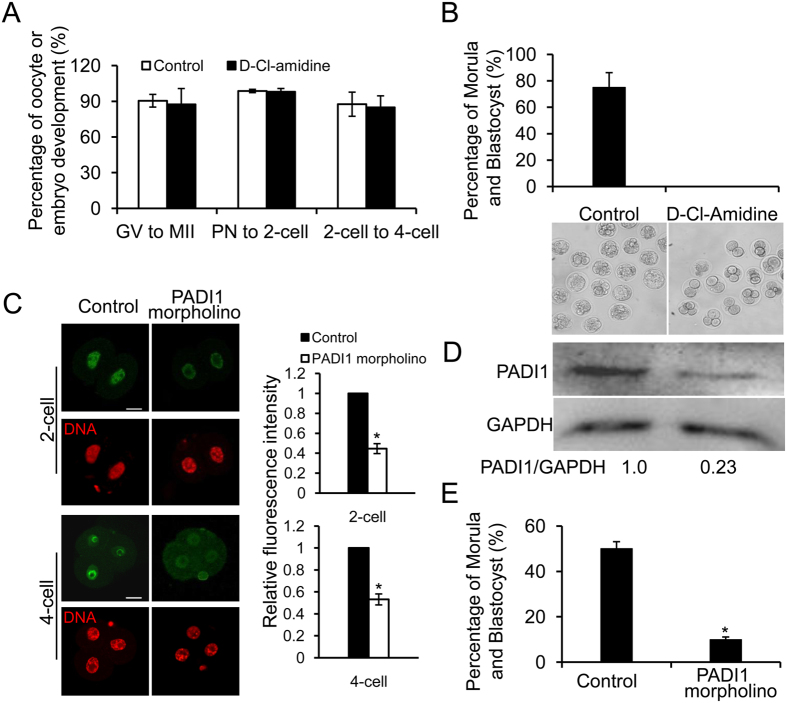
Inhibiting PADI1 activity or ablation of PADI1 blocks embryonic development *in vitro* beyond 4-cell stage. (**A**) D-Cl-amidine treatment did not affect the rate of oocyte maturation (n = 285, the controls and n = 239, D-Cl-amidine treatment), zygote to 2-cell (n = 339, the controls and n = 342, D-Cl-amidine treatment), and 2-cell to 4-cell transitions (n = 402, the controls and n = 330, D-Cl-amidine treatment). (**B**) Pronuclear stage zygotes were cultured in KSOM medium supplemented without (n = 392) or with 100 μM D-Cl-amidine (n = 357) and no embryo developed to the morula or blastocyst stage. The lower panel showing the representative embryos observed by light microscopy. (**C**) Immunostaining confirmed that nuclear PADI1 expression significantly decreased in Padi1-MO-injected embryos at the 2- or 4-cell stage compared to the control embryos (P < 0.05). DNA was counterstained with DAPI. Scale bar, 20 μm. (**D**) The efficiency of Padi1-MO was verified by Western blot. Band intensity was calculated using ImageJ software, and the ratio of PADI1/GAPDH expression was normalized. (**E**) Depletion of PADI1 led to developmental arrest. PN zygotes were injected with Padi1-MO (n = 81) or Control-MO (n = 80), and cultured until the blastocyst stage. The embryos from each experimental group were counted and scored according to their developmental stage. Shown is the percentage of morula and blastocyst embryos from three independent experiments. ^*^P < 0.05.

We next wished to assess the function of PADI1 at the beginning of the mouse embryo development. To this end, we used two methods: inhibition of PADI1 enzyme activity and injection of Padi1-targeting morpholino (Padi1-MO). Previous studies have shown that inhibiting PADI activity in the mouse early embryos using 250 μM of the pan-PADI inhibitor, Cl-amidine, blocked early cleavage divisions *in vitro,* with embryos mainly arrested at the 2- and 4-cell stages^21^. We found that specifically inhibiting PADI1 activity with 100 μM of the PADI1-specific inhibitor D-Cl-amidine, a next generation Cl-amidine analogue^22^, did not affect oocyte maturation or pronuclear formation. Zygotes cultured under 100 μM D-Cl-amidine successfully proceeded through the first two cleavages and reached the 4-cell stage, at the same time as that in the phosphate-buffered saline (PBS) control group (Fig. 2A). However, no zygotes were found to have progressed beyond the 4-cell stage of development (0%, n = 357) while 75% of embryos (n = 392) cultured in KSOM media supplemented with PBS developed to the morula or blastocyst (Fig. 2B and Table 1). To exclude the possibility that D-Cl-amidine blocked development is due to a non-specific toxic side-effect, we examined embryo viability following D-Cl-amidine treatment using the vital dye propidium iodide (PI). PI is excluded by viable cells but can penetrate cell membranes of dying or dead cells to intercalate into double-stranded nucleic acids. PN zygotes were cultured for 68 hours in KSOM medium supplemented with 100 μM D-Cl-amidine or PBS. Embryos were then fixed and mounted on glass slides for confocal laser scanning microscope analysis. Results showed that nuclei from D-Cl-amidine treated (arrested at 4-cell stage) or PBS treated embryos (developed to 8-cell stage) were not stained with PI while nuclei from D-Cl-amidine treated embryos followed by 0.1% Triton extraction were strongly stained with PI (Supplementary Fig. S2). These results suggest that D-Cl-amidine treatment does not affect cell membrane health, therefore early embryo development arrest is likely due to the specific inhibition of PADI1 activity.

For depletion of PADI1, we injected Padi1-MO into the zygotes. A non-targeting MO was injected as a negative control. A significant reduction of PADI1 protein level in the 2- and 4-cell embryos was confirmed by immunofluorescence staining (P < 0.05) (Fig. 2C) and western blotting (Fig. 2D). We found that the majority of the embryos injected with Padi1-MO arrested at the 4-cell stage (82.72%, n = 81), with only 9.87% (n = 81) of the embryos developing beyond the morula and blastocyst stages. In sharp contrast, 50% (n = 80) of the control embryos developed beyond the morula stage (Fig. 2E and Table 2). Thus, injection of Padi1-MO, similarly to PADI1 inhibition, caused the majority of embryos to arrest at the 4-cell stage. Together, these findings suggest that PADI1 activity is required for progression of embryonic development beyond the 4-cell stage.

### Inhibiting PADI1 or depletion of PADI1 suppresses citrulline levels on histone tails in early embryos

**Figure 3:**
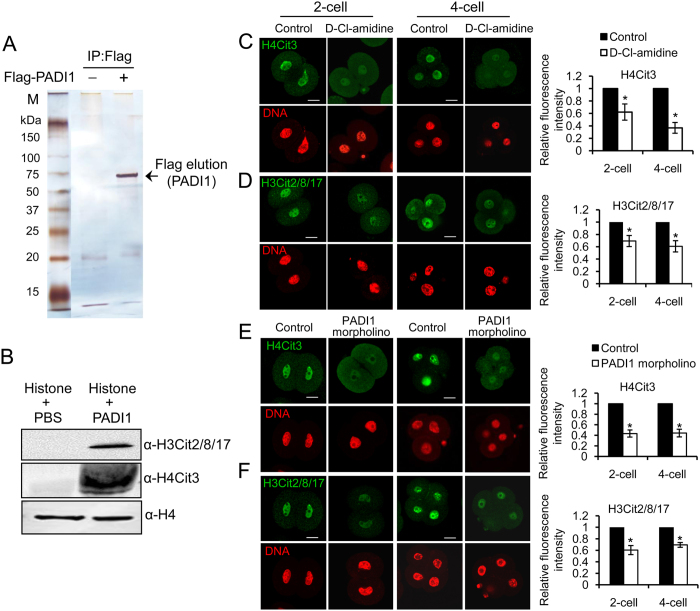
Inhibiting or knockdown PADI1 decreases histone citrullination in mouse 2-cell and 4-cell stage of embryos. (**A**) Silver staining of the Flag-tagged PADI1 protein purified from Flag-PADI1 overexpressed HEK293 cells. (**B**) Western blotting showing the anti-H3Cit2/8/17 and anti-H4Cit3 antibodies specifically reactive with the appropriately sized band from PADI1-treated bulk histones but not from nontreated histones. Anti-histone H4 staining revealed the presence of approximately equal amounts of histone in each lane. (**C–F**) PN zygotes were cultured in KSOM medium for 24 h (2-cell stage) and 48 h (4-cell stage) supplemented with 100 μM D-Cl-amidine or equal volume of PBS as a control. Embryos were collected and fixed for further immunofluorescence staining with anti-H3Cit2/8/17 and anti-H4Cit3 antibodies, respectively. DNA was counterstained with DAPI. Scale bar, 20 μm. Representative images and quantification of the immunofluorescence signals from each group are shown. At least 40 embryos at each stage for each group were analyzed, and the experiments were repeated 3 times. Error bars indicate mean ± SD. *P < 0.05 vs. controls.

A previous report has demonstrated the robust citrullination of histone H3 and H4 tails from 2-cell to blastocyst stage during preimplantation development^21^. Given that PADI1 is the dominant nuclear PADI in early embryos, and that PADI1 showed similar spatiotemporal expression pattern with that of the histone tail citrullination during preimplantation development, we asked whether PADI1 catalyzes histone tail citrullination in the early embryos. To this end, we first confirmed that PADI1 targeted histone tails for citrullination *in vitro*. Purified Flag-tagged PADI1 protein from Flag-PADI1 overexpression HEK293 cells (Fig. 3A) was used to citrullinate bulk histones. Immunoblotting the resolved histones with H3Cit2/8/17 and H4Cit3 antibodies showed that these two antibodies were reactive with an appropriately sized band from the PADI1-citrullinated histones but was not reactive with untreated histones (Fig. 3B).

Next, we tested whether D-Cl-amidine suppressed citrulline levels on histones in early embryos using the H3Cit2/8/17 and H4Cit3 antibodies. PN zygotes were cultured in KSOM media supplemented without or with 100 μM D-Cl-amidine. Embryos collected at the 2- and 4-cell stages were fixed, stained with the anti-citrullinated histone antibodies and then evaluated by laser scanning confocal microscopy. Results showed that staining levels for the H4Cit3 and H3Cit2/8/17 antibodies were reduced significantly compared to the KSOM control group (P < 0.05) (Fig. 3C,D). To further test whether histone citrullination could be eliminated by higher concentration of D-Cl-amidine, we cultured PN zygotes in KSOM media supplemented with 200 uM D-Cl-amidine and collected at the 4-cell stage for the following immunostaining. Results showed that the H3Cit2/8/17 and H4Cit3 staining signals were reduced to undetectable levels compared to the KSOM control group (Supplementary Fig. S3). Similarly, injection of the Padi1-MO into zygotes also reduced histone H4Cit3 and H3Cit2/8/17 levels at 2- and 4-cell stages (Fig. 3E,F). These results strongly suggest that PADI1 can catalyze histone H3 and H4 tail citrullination in early embryos, which may facilitate gene expression in preimplantation development.

### Ablation of PADI1 function leads to a dramatic decrease of genome transactivation

**Figure 4:**
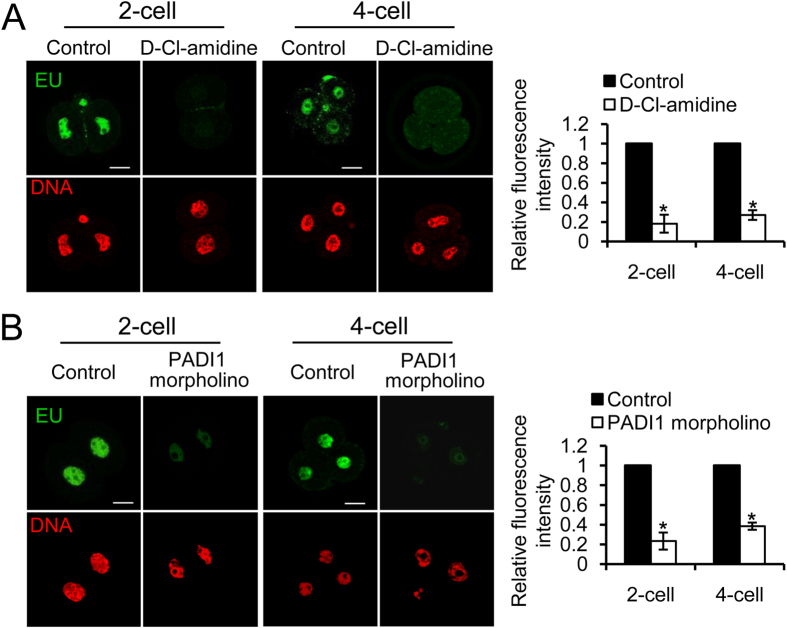
Immunofluorescence staining for EU incorporation. Representative confocal images demonstrating a significant decrease of EU positive nuclear signals in the 2- or 4-cell stage of embryos treated with D-Cl-amidine (**A**) or Padi1-MO injection (**B**), compared with the control embryos, respectively. DNA was stained with Hoechst 33342 (red). Scale bar, 20 μm. Histogram on the right panel showing the quantification of EU incorporation data. Error bars indicate mean ± SD from 3 independent experiments. *P < 0.05 vs. controls.

Changes in chromatin structure are thought to play an important role in reprogramming gene expression during preimplantation development^23^,^24^,^25^. We postulated that the observed decrease in histone citrullination in PADI1-depleted or D-Cl-amidine-treated embryos could inhibit genome transactivation at 2–4 cell stages, thus leading to early embryo development arrest. To test his hypothesis, we exploited 5-ethynyl uridine (EU) incorporation in living cells followed by “click chemistry” to detect populations of newly synthesized RNA^26^. EU, an alkyne-modified nucleotide, when incubated with the live cells, can be actively incorporated into nascent RNA. Therefore, PN zygotes were cultured in KSOM media supplemented without or with 100 μM D-Cl-amidine. Before embryo collection, 1 mM EU was added to the culture medium and incubated for another 3 hours. Then embryos at 2- and 4-cell stages were collected and fixed, followed by immunofluorescence analysis. As shown in Fig. 4, EU incorporation was readily detected in the early embryos in the control groups. However, D-Cl-amidine treatment or Padi1-MO injection significantly decreased EU incorporation, indicating a decrease of overall transcriptional activity (P < 0.05). It has been documented that several stage-specific genes involved in embryo developmental are actively transcribed after 4-cell stages^3^. Therefore, we further analyzed the expression of some of these genes in both control and D-Cl-amidine treated 4-cell embryos. Compared with the control embryos, D-Cl-amidine treated embryos displayed significantly decreased expression of Pou5f1. Expression of the rest of the genes (Eomes, Crtr-1, Utf1) was not significant as a result of the large variation, but the expression was still obviously lower in D-Cl-amidine treated embryos (Supplementary Fig. S4), further confirming that PADI1-enzyme activity is required for genome transactivation at 2–4 cell stages.

### Hypophosphorylation of RNA Pol II Ser2 in D-Cl-amidine-treated or PADI1-depleted 2- and 4-cell early embryos

**Figure 5:**
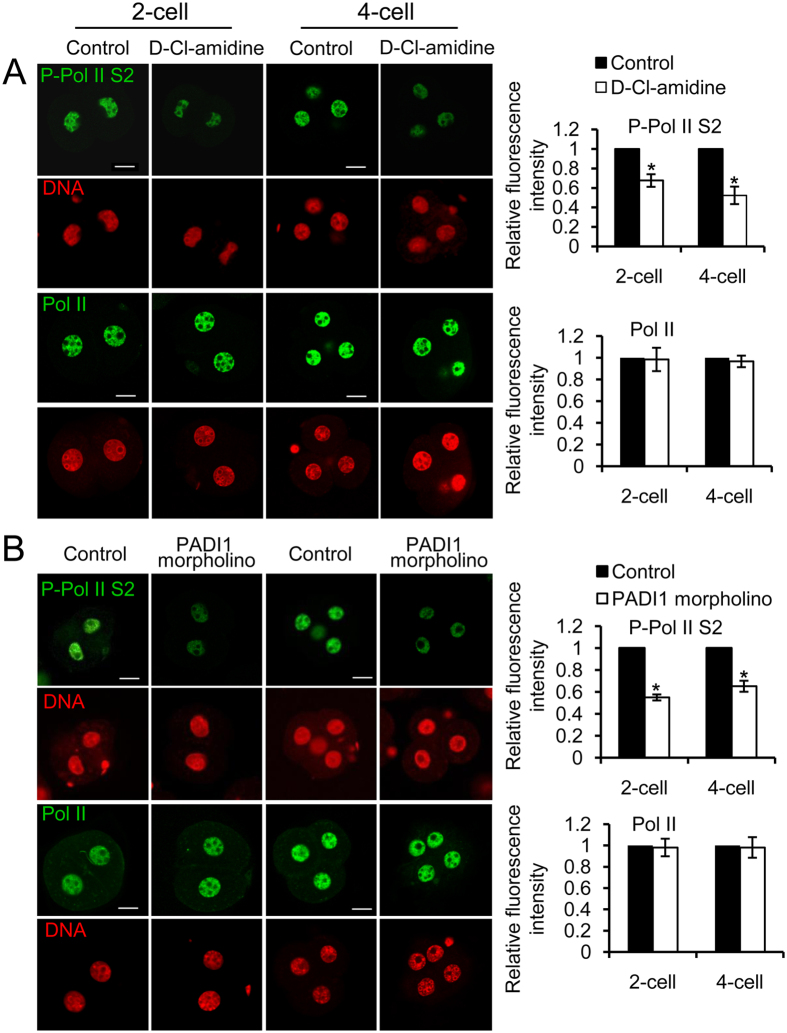
Effect of inhibition of PADI1 or PADI1-knockdown on RNA Pol II serine 2 phosphorylation in mouse 2- and 4-cell stage embryos. D-Cl-amidine-treated (**A**), and Padi1-MO injected (**B**) embryos (collected at the 2-cell stage and 4-cell stage separately) were immunostained with anti-RNA Pol II Ser2 (P-Pol II S2) and anti-RNA Pol II antibodies, and co-stained with DAPI for DNA. Scale bar, 20 μm. Representative images and quantification of the immunofluorescence signals from each group are shown. At least 40 embryos at each stage for each group were analyzed, and the experiments were repeated 3 times. Error bars indicate mean ± SD. *P < 0.05 vs. controls.

Phosphorylated (Ser2) RNA polymerase II (RNA Pol II) is a well-characterized marker for active transcription, and has been proposed as a landmark of zygotic gene activation in early embryos^27^. To gain more insight into the molecular mechanism underlying the dramatic decrease in genome transactivation in 2- and 4-cell stage of embryos upon inhibition or depletion of PADI1, we analyzed whether RNA Pol II Ser2 phosphorylation levels in these arrested embryos were affected. Results showed a statistically significant, ~30–40% reduction of RNA Pol II phosphorylation at Ser2 in D-Cl-amidine-treated embryos at 2- and 4-cell stages (P < 0.05). However, this treatment did not seem to change the total level of RNA Pol II (Fig. 5A). As expected, knockdown of Padi1 through Padi1-MO injection also significantly decreased the RNA Pol II phosphorylation at both stages (P < 0.05) (Fig. 5B). Together, these results further confirmed our hypothesis that inhibiting PADI1 activity or knockdown of Padi1 in 2- or 4-cell stage of embryos reduced histone H4Cit3 and H3Cit2/8/17, which may in turn disturb the local chromatin conformation, contributing to, at least in part, the hypophosphorylation of RNA Pol II Ser2 and resulting in the decrease of overall transcriptional activity in preimplantation development.

## Discussion

Multiple posttranslational modifications of histone tails have been identified as the key epigenetic marks to correlate with gene expression patterns during the preimplantation development transitions, including acetylation, methylation, ubiquitination^28^,^29^,^30^,^31^. These modifications influence gene transcriptional outcome by modulating local chromatin structure, allowing a more permissive chromatin state, and facilitating transcription^32^. More recently, histone citrullination of histone H4R3 and H3R2/8/17 has also been found robustly activated in the nuclei in preimplantation embryos, suggesting that PADI-mediated histone citrullination may play an important role in early development^21^. However, it was not clear which PADI catalyzed these modifications. We first excluded PADI6 since PADI6 does not contain enzyme activity^15^. We next excluded the possibility of PADI2 and PADI4, as H4Cit3 and H3cit2/8/17 did not show any appreciable loss in these two mutant mouse lines^15^ (Supplementary Fig. S5). Studies from Brahmajosyula *et al*.^33^ has detected nuclear PADI4 and citrullinated histone H3 in mouse embryos, this discrepancy could be caused by the two different anti-PADI4 antibodies used. We are currently generating Padi1-null mouse lines and future studies on the histone citrullination in Padi1 KO mouse oocytes and early embryos will help confirm the enzyme activity of PADI1. Given that PADI1 is the only PADI with strong nuclear staining from the 2-cell to blastocyst stage, and that PADI3 was not found in the nuclei of early embryos until the morula stage (Fig. 1), it seems likely that PADI1, but not the other PADIs, may catalyze these specific citrulline modifications on histone tails in early embryos. To further confirm our hypothesis, we have provided two lines of evidence: First, zygotes treated with D-Cl-amidine dramatically inhibited histone H3 and H4 citrullination in the 2- and 4-cell stage embryos (Fig. 3C,D). Second, injection of Padi1-MO into the zygotes reduced endogenous PADI1 (Fig. 2C,D) thus leading to the decreased histone H4Cit3 and H3Cit2/8/17 (Fig. 3E,F) at these two stages. Together, we propose that PADI1 localizes in mouse early embryos and contributes to histone H3 and H4 tail citrullination.

Unlike the other PADIs, studies on PADI1 are very limited. PADI1 is expressed in a few tissues, including the epidermis and uterus, where it is thought to promote the differentiation of keratinocytes by citrullinating the intermediate filament, keratin, and filaggrin^34^,^35^,^36^,^37^. However, little else is known regarding the normal and pathological roles of PADI1 in other tissues. This is the first report showing that PADI1 is expressed in early embryos and appears to mediate histone citrullination during preimplantation development. Regarding the role of histone citrullination on preimplantation development, a previous study has shown that inhibiting histone citrullination prevented embryo development beyond the 4-cell stage^21^. Our studies further demonstrate that it is PADI1 that mediates this modification in the early embryos, and PADI1-catalyzed histone citrullination is vital to early embryo progression beyond the 4-cell stage.

During preimplantation development, programmed waves of gene activation may serve to satisfy the developing embryo’s requirement for specific classes of products at specific times. For example, ZGA transcripts and their protein products seem to be required for the progression of embryos beyond the 4-cell stage^2^,^38^. Given that histone citrullination of histone H3 and H4 correlates well with gene transactivation^16^,^17^,^18^,^19^,^20^, and histone citrullination is robustly activated in the nuclei in preimplantation embryos^21^, it seems likely that decreased histone citrullination on H3 and H4 at the 2- and 4-cell stages, due to completely inhibiting or depleting PADI1, may disturb an open chromatin structure, prevent a more permissive chromatin state, and inhibit the gene activation required for embryo development, thus leading to developmental arrest at the 4-cell stage. Studies are currently underway to further identify global gene expression patterns regulated by PADI1 in the early embryos and will fully dissect the mechanism by which PADI1 regulates gene expression during mouse preimplantation development.

## Materials and Methods

All chemicals and reagents were obtained from Sigma unless otherwise stated. Animal care and experimental procedures were approved by the Animal Care and Use Committee of Nanjing Medical University. The study protocol was carried out in accordance with institutional guidelines. D-Cl-amidine was synthesized as previously described^22^.

### Collection of oocytes, early embryos, and culture of zygotes

Female ICR mice (4–6 weeks) were injected intraperitoneally with 10 international units (IU) of pregnant mare serum gonadotropin (PMSG). Forty-eight hours later, the mice were sacrificed and the ovaries were isolated and extensively punctured with two 30-gauge needles. Immature oocytes displaying germinal vesicle (GV) were collected from ovaries in M2 medium. For collection of MII oocytes, 6–8-week-old female ICR mice were injected intraperitoneally with 10 IU human chorionicgonadotropin (hCG) 48 hours post PMSG injection. 16 hours later, MII oocytes were collected after digestion with 0.1% hyaluronidase.

To obtain fertilized oocytes and preimplantation embryos, female mice were mated with 10–12-week-old males. Successful mating was confirmed by the presence of vaginal plugs. Zygotes, 2-cell, 4-cell, 8-cell, morula, and blastocyst stage embryos were collected 20, 42, 55, 67, 80, and 96 hours post hCG injection, separately.

For early embryos culture, zygotes were collected 20 hours post hCG injection from the ampulla oviducts of superovulated females that had been mated with 10–12-week-old males just after injection of hCG. Surrounding cumulus cells were dispersed by treatment with 0.1% hyaluronidase for 5 min. Cumulus-free zygotes were washed with M2 medium. Embryos were transferred and cultured in fresh KSOM medium (Millipore, MR-020P-D) under mineral oil in a 5% CO2 incubator at 37 °C for subsequent development. Then, 2-cell, 4-cell, 8-cell, morula, and blastocyst stage embryos were collected after 24, 48, 64, 76, and 100 hours of culture, respectively.

### Inhibitor Treatment

D-Cl-amidine was dissolved in PBS. GV oocytes were cultured in M2 medium for 14 hours (MII oocytes) supplemented with 100 μM D-Cl-amidine or equal volume of PBS. PN zygotes were cultured in KSOM medium supplemented with 100 μM D-Cl-amidine or equal volume of PBS for indicated hours as states above.

### Morpholino (MO) microinjection and *in vitro* culture

PADI1-targeting morpholino (5′-GTCGAGCTTCCAGTCTCCTGGTC-3′) was purchased from Gene Tools LLC (Philomath, OR, USA), and then diluted with water to give a working concentration of 1 mM. About 5 pl morpholino solution was microinjected into the zygotes obtained as described above using a Narishige microinjector (Tokyo, Japan). A non-targeting MO was injected as a control. After microinjection, zygotes cultured in fresh KSOM medium under mineral oil in a 5% CO2 incubator at 37 °C for subsequent development.

### Immunofluorescent and confocal microscopy

Oocytes and embryos were fixed in 4% paraformaldehyde in PBS (pH 7.4) for 30 min and then permeabilized in 0.5% Triton-X-100 for 30 min at room temperature. After 1 hour blocking in 1% BSA/PBS at room temperature, samples were incubated overnight at 4 °C with primary antibodies as following: anti-PADI1 at 1:100 (Cat# HPA028133, Sigma), anti-PADI2 at 1:100 (Cat# 12110-1-AP, Proteintech), anti-PADI3 at 1:100 (Cat# ab50246, Abcam), anti-PADI4 at 1:100 (Cat# P4749, Sigma), anti-H4Cit3 at 1:50 (Cat# 07-596, Millipore), anti-H3Cit2/8/17 at 1:100 (Cat#ab77164, Abcam), anti-P-Pol II S2 at 1:100 (Cat# ab193468, Abcam), anti-Pol II at 1:100 (Cat# sc-12823, Santa Cruz). After five washes, samples were incubated with FITC-conjugated secondary antibody for 1 hour at room temperature. Nuclei were counterstained with DAPI for 5 min. Oocytes and embryos were mounted on glass slides in a drop of antifade medium (Vectashield, Burlingame, CA, USA) and examined under confocal laser scanning microscope (LSM 700; Zeiss, Oberkochen, Germany). Image J software (National Institutes of Health, Bethesda, MD, USA) was used to quantify fluorescence intensity. Fluorescence intensity was randomly measured in at least five regions of interest strictly limited to the nucleus of each embryo as stated previously^39^. Fluorescence signal was calculated as the average intensity after background subtraction.

### Analysis of embryo viability

PN zygotes obtained as described above were cultured for 68 hours in KSOM medium supplemented with 100 μM D-Cl-amidine or equal volume of PBS (controls). Embryos were then incubated with 20 μg/ml PI in KSOM for 5 min at 37 °C in a 5% CO_2_ incubator. After 3 washes, embryos were fixed in 4% paraformaldehyde in PBS for 30 minutes. Finally, oocytes were mounted on glass slides and examined under confocal laser scanning microscope. A subset of D-Cl-amidine-treated embryos were permeabilized with 0.1% Triton for 20 min prior to PI staining to serve as positive controls.

### EU incorporation assays

EU incorporation assays were performed using Click-iT RNA Imaging kits (C10329, Invitrogen). PN zygotes were cultured for 21 hours (2-cell embryos) or 45 hours (4-cell embryos) in KSOM medium supplemented with 100 μM D-Cl-amidine or equal volume of PBS (controls). Then 1 mM 5-ethynyl uridine (EU) was added to the culture for another 3 hours of incubation prior to Hoechst 33342 staining according to kit instructions. For morpholino-injected embryo culture, PN zygotes injected with Padi1-MO or control-MO were cultured for 21 hours (2-cell embryos) or 45 hours (4-cell embryos) in KSOM medium, followed by the same EU treatment as above. Images were captured using confocal lasers canning microscope. Fluorescence intensity was measured as described above. Fluorescence signal was calculated as the average intensity after background subtraction.

### Gene-Specific Expression Analyses

Total RNA was isolated from embryos using Qiagen RNeasy Mini Kit in combination with on-column DNase treatment (ABI). High Capacity RNA-to-cDNA kit (ABI) was used to synthesize the first strand of cDNA. Quantitative real-time PCR (qPCR) was performed using Power SYBR Green PCR Master Mix (ABI) with gene-specific primers. All target gene transcripts were normalized to Gapdh. The primers used for the qPCR are Pou5f1 Forward: 5′-AGAGGATCACCTTGGGGTACA-3′, Reverse: 5′-CGAAGCGACAGATGGTGGTC-3′; Eomes Forward: 5′-GGCCCCTATGGCTCAAATTCC-3′, Reverse: 5′-GAACCACTTCCACGAAAACATTG-3′; Crtr-1 Forward: 5′-CAGCCCGAACACTACAACCAG-3′, Reverse: 5′-CAGCCGGATTTCATACGACTG-3′; Utf1f Forward: 5′-CCGGACCCTTCGATAACCAG-3′, Reverse: 5′-CAGAGTGTCGGTGCTCGTAA-3′; Gapdh Forward: 5′-CTTTGTCAAGCTCATTTCCTGG-3′, Reverse:5′-TCTTGCTCAGTGTCCTTGC-3′.

### Cell culture and transfection

HEK293 cells were maintained in DMEM supplemented with 10% fetal bovine serum. Flag-tagged PADI1, PADI2, and PADI4 in pcDNA3.1 (+) or His-tagged PADI1, PADI3 in pcDNA3.1 (+) plasmids including the empty control vectors were transfected into 293 cells using FuGENE 6 following the manufacture’s instruction. The cells were collected and lysed 48 hours post transfection for the following western blotting assays.

### Immunoprecipitation and silver staining

Flag-tagged PADI1 plasmids were transiently transfected into HEK293 cells using Fugene6 (Roche). The cells were collected and lysed 48 hours post transfection. The whole-cell lysates were immunoprecipitated with anti-Flag M2 affinity gel (#A2220, Sigma) following the manufacture’s instruction. Elution of Flag fusion proteins (PADI1) were separated by 10% polyacrylamide gel electrophoresis (SDS-PAGE), followed by silver staining using SilverXpress® Silver Staining Kit (#LC6070, Invitrogen).

### Acid extraction of histones, PADI assay and western blotting

Cellular histones were purified by acid extraction and the PADI assay was performed as described previously^21^. Briefly, bulk histones were treated with Flag-tagged PADI1 in PADI buffer containing 50 mM Tris-HCl (pH 7.6), 4 mM DTT, 4 mM CaCl_2_ at 37 °C for 1 hour. Histone samples were separated by 15% SDS-PAGE and electrically transferred to PVDF membrane. The membranes were blocked in 5% milk diluted by TBST for 1 hour at room temperature and then incubated overnight at 4 °C with anti-H4Cit3 (1:1000), anti-H3Cit2/8/17 (1:1000) and anti-H4 antibody (1:1000), seperately. After 3 washes in TBST, the membranes were incubated for 1 hour with horseradishperoxidase (HRP)-conjugated secondary antibody. Specific proteins were detected using ECL Plus Western Blotting Detection System (GE Healthcare, Piscataway, NJ, USA).

### Statistics

Each experiment was repeated at least three times. All data are presented as mean ± SD, unless otherwise indicated. Statistical comparisons were made using Student’s t-test. P < 0.05 was considered to be significant.

## Additional Information

**How to cite this article**: Zhang, X. *et al*. Peptidylarginine deiminase 1-catalyzed histone citrullination is essential for early embryo development. *Sci. Rep.* **6**, 38727; doi: 10.1038/srep38727 (2016).

**Publisher's note:** Springer Nature remains neutral with regard to jurisdictional claims in published maps and institutional affiliations.

## Supplementary Material

Supplementary Information

## Figures and Tables

**Table 1:** The effect of D-Cl-amidine on early embryonic development.

Group	No.	Stage of embryos (%)		
		≤4-cell	8-cell	≥Morula
Control	392	88 (22.45%)	10 (2.55%)	294 (75.0%)
D-Cl-amidine	357	357 (100%)	0	0

**Table 2:** The effect of Padi1-MO injection on early embryonic development.

Group	No.	Stage of embryos (%)		
		≤4-cell	8-cell	≥Morula
Control	80	33 (41.25%)	7 (8.75%)	40 (50.0%)
Padi1-MO	81	67 (82.72%)	6 (7.41%)	8 (9.87%)
