# Supplementary material for: Peptidylarginine deiminase 1-catalyzed histone citrullination is essential for early embryo development
Source: Sci Rep. 2016 Dec 8;6:38727. doi: 10.1038/srep38727 (PMC5144008; doi:10.1038/srep38727)
Supplement: Supplementary Information [file srep38727-s1.pdf]

Supplementary information

Peptidylarginine deiminase 1-catalyzed histone citrullination is essential for early embryo development

Xiaoqian Zhang [1,*], Xiaoqiu Liu[2,3,*], Mei Zhang[1], Tingting Li[1], Aaron Muth,[4] Paul R. Thompson[4] , Scott A. Coonrod[5] & Xuesen Zhang[1]

[1]State Key Laboratory of Reproductive Medicine, Nanjing Medical University, Nanjing, China.
[2]Key Laboratory of Pathogen Biology of Jiangsu Province, Nanjing Medical University, Nanjing, China
[3]Department of Microbiology, Nanjing Medical University, Nanjing, China
[4]Department of Biochemistry and Molecular Pharmacology, University of Massachusetts Medical School, Worcester, MA, USA
[5]Baker Institute for Animal Health, College of Veterinary Medicine, Cornell University, Ithaca, NY, USA
[*]These authors contributed equally to this work.

Correspondence and requests for materials should be addressed to:
Xuesen Zhang
E-mail: xuesenzhang@njmu.edu.cn

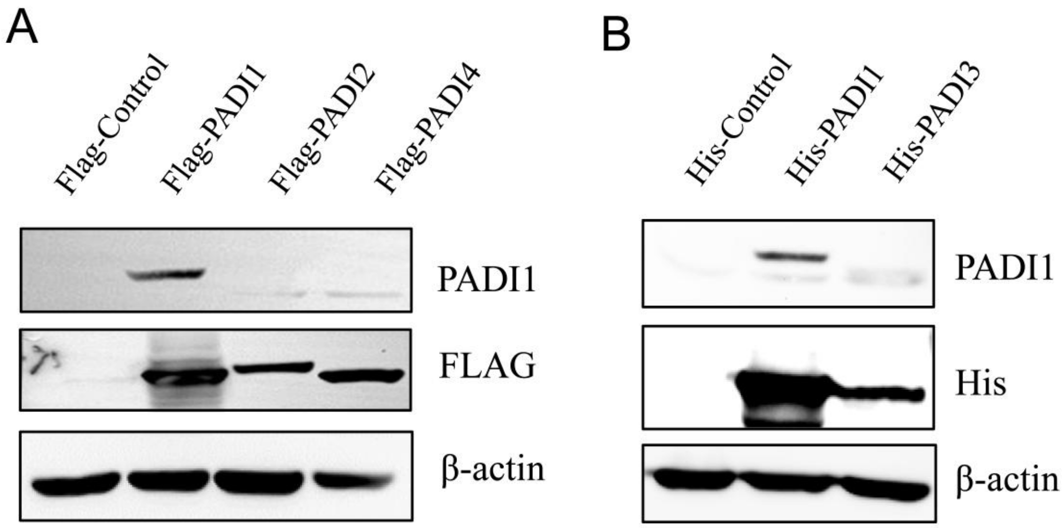

Supplementary Figure 1. PADI1 antibody specificity. (A) Western blot of HEK293 cell extracts following transfection of either Flag-pcDNA3.1 (+) alone or pcDNA3.1 containing the Flag-tagged PADI1, PADI2, PADI4 plasmids. The PADI1-Flag fusion protein was detected using an anti-PADI1 antibody. Anti-Flag antibody was used to show the equal amount of PADI isoform expressed in the cells, and β-actin was used for loading control. **(B)** Western blot of HEK293 cell extracts following transfection of either His-pcDNA3.1 or pcDNA3.1 containing the His-tagged PADI1, PADI3 plasmids. The PADI1-His fusion protein was detected using an anti-PADI1 antibody. Anti-His antibody was used to show the equal amount of PADI isoforms expressed in the cells, and β-actin was used for loading control.

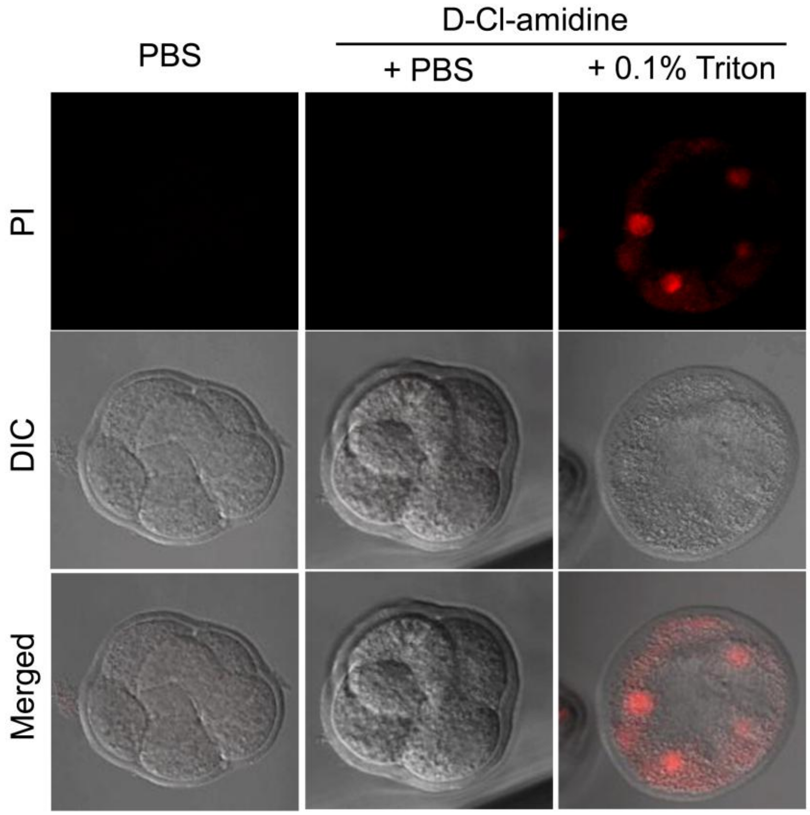

Supplementary Figure 2. Assessment of embryo viability following D-Cl-amidine treatment. Pronuclear stage zygotes were cultured for 68 hours in KSOM medium supplemented with 100 μM D-Cl-amidine or PBS (control) prior to the staining. Embryos were stained with 20 μg/ml of PI in KSOM for 5 min and images were recorded by confocal laser scanning microscope. A subset of D-Cl-amidine treated embryos were permeablized with 0.1% Triton-X100 for 20 min prior to PI staining to serve as positive controls to show the nuclear staining of nonviable cells. PI, Propidium iodide. DIC, differential interference contrast.

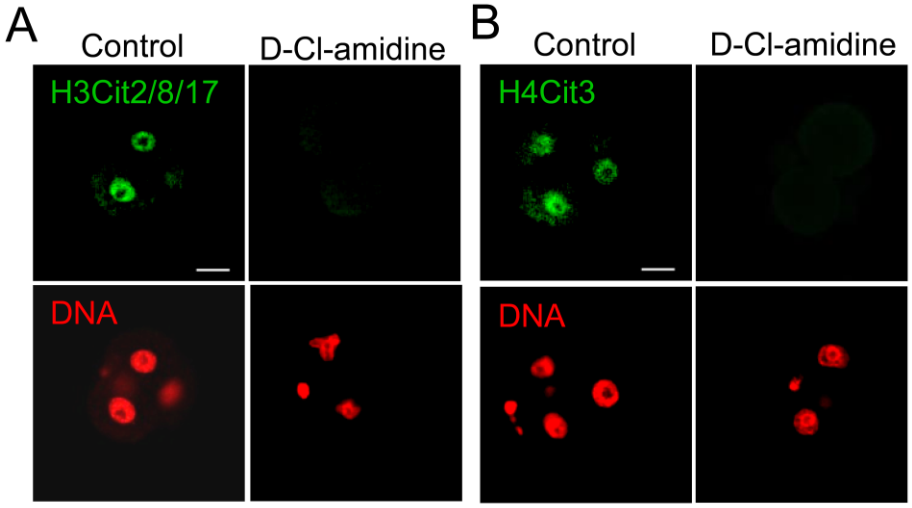

Supplementary Figure 3. Inhibiting PADI1 with 200 uM D-Cl-amidine eliminates histone citrullination in 4-cell stage of embryos. PN zygotes were cultured in KSOM medium for 48 h (4-cell stage) supplemented with 200 μM D-Cl-amidine or equal volume of PBS as a control. Embryos were collected and fixed for further immunofluorescence staining with anti-H3Cit2/8/17 **(A)** and anti-H4Cit3 **(B)** antibodies, respectively. DNA was counterstained with DAPI. Scale bar, 20 μm.

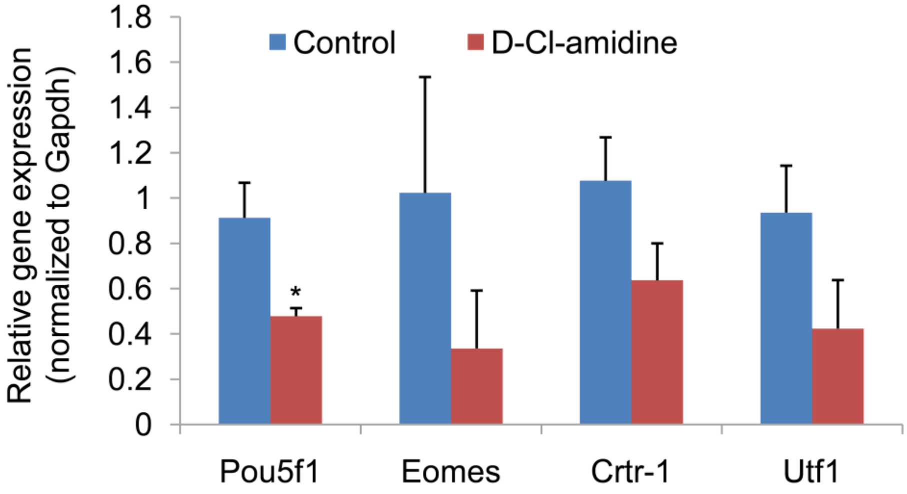

Supplementary Figure 4. Comparison of the relative expression of genes involved in transactivation in normal 4-cell embryos and embryos treated with D-Cl-amidine. Embryos treated with D-Cl-amidine displayed significant decreased expression of Pou5f1 compared to the PBS controls. Expression of the rest of the genes (Eomes, Crtr-1, Utf1) was not significant as a result of the large variation, but the expression was still obviously lower in D-Cl-amidine treated embryos. [*]P<0.05.

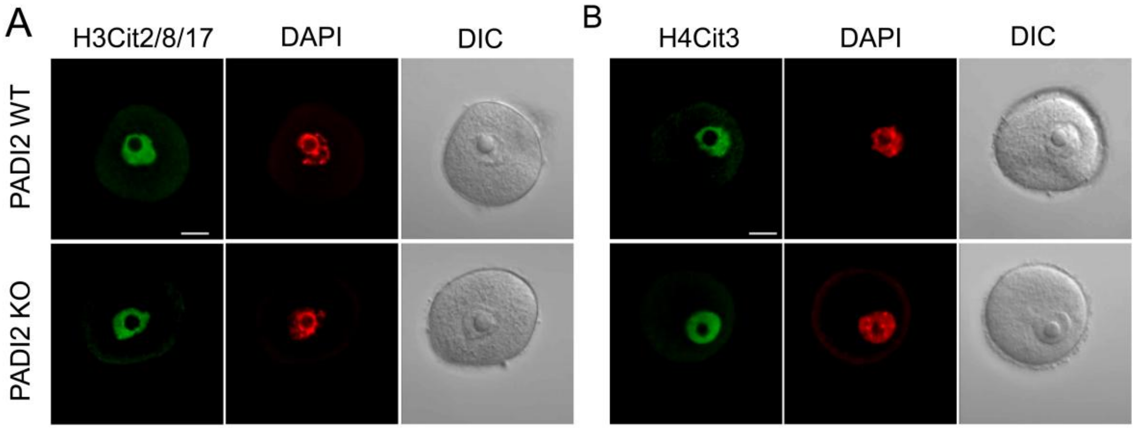

Supplementary Figure 5. Comparison of citrullination levels at H3R2/8/17 and H4R3 in PADI2 wild-type and null oocytes. Confocal images were taken of wild-type and PADI2 null GV stage oocytes that had been probed with antibodies to H3Cit2/8/17 **(A)** and H4Cit3 **(B)**. Oocytes were counterstained with DAPI to visualize DNA. DIC, differential interference contrast.
